# Microbial Activities and Dissolved Organic Matter Dynamics in Oil-Contaminated Surface Seawater from the Deepwater Horizon Oil Spill Site

**DOI:** 10.1371/journal.pone.0034816

**Published:** 2012-04-11

**Authors:** Kai Ziervogel, Luke McKay, Benjamin Rhodes, Christopher L. Osburn, Jennifer Dickson-Brown, Carol Arnosti, Andreas Teske

**Affiliations:** 1 Department of Marine Sciences, University of North Carolina at Chapel Hill, Chapel Hill, North Carolina, United States of America; 2 Department of Marine, Earth and Atmospheric Sciences, North Carolina State University, Raleigh, North Carolina, United States of America; University of California Merced, United States of America

## Abstract

The Deepwater Horizon oil spill triggered a complex cascade of microbial responses that reshaped the dynamics of heterotrophic carbon degradation and the turnover of dissolved organic carbon (DOC) in oil contaminated waters. Our results from 21-day laboratory incubations in rotating glass bottles (roller bottles) demonstrate that microbial dynamics and carbon flux in oil-contaminated surface water sampled near the spill site two weeks after the onset of the blowout were greatly affected by activities of microbes associated with macroscopic oil aggregates. Roller bottles with oil-amended water showed rapid formation of oil aggregates that were similar in size and appearance compared to oil aggregates observed in surface waters near the spill site. Oil aggregates that formed in roller bottles were densely colonized by heterotrophic bacteria, exhibiting high rates of enzymatic activity (lipase hydrolysis) indicative of oil degradation. Ambient waters surrounding aggregates also showed enhanced microbial activities not directly associated with primary oil-degradation (β-glucosidase; peptidase), as well as a twofold increase in DOC. Concurrent changes in fluorescence properties of colored dissolved organic matter (CDOM) suggest an increase in oil-derived, aromatic hydrocarbons in the DOC pool. Thus our data indicate that oil aggregates mediate, by two distinct mechanisms, the transfer of hydrocarbons to the deep sea: a microbially-derived flux of oil-derived DOC from sinking oil aggregates into the ambient water column, and rapid sedimentation of the oil aggregates themselves, serving as vehicles for oily particulate matter as well as oil aggregate-associated microbial communities.

## Introduction

The explosion of the Deepwater Horizon drilling rig in the northern Gulf of Mexico on April 20, 2010, resulted in the largest accident in the history of the U.S. petroleum industry [1]. Oil release in water depths of around 1500 m during the 84 days of the spill was estimated at 4.1 million barrels [2]. Plumes enriched in water-soluble components of the spilled petroleum hydrocarbons developed in water depths greater than 1000 m within two weeks after the onset of the spill [3], [4]. These deep water plumes were found to contain high microbial biomass dominated by heterotrophic bacteria capable of degrading petroleum hydrocarbons [5]–[10]. Oil-degrading bacteria play a key role in the biodegradation of crude oil in the ocean [11], and high activities of microbial oil degradation in the deep water plume possibly caused the observed decline of petroleum hydrocarbons in deep waters around the wellhead within months after the onset of the spill [2].

An estimated 60% of the oil released by the riser pipe in the deep sea ultimately reached the sea surface [1] where chemical weathering (evaporation, photoxidation) and hydrodynamic forces (wind-driven surface waves and currents) affected the distribution and properties of the crude oil [12]. For example, on-site observations during sampling revealed that weathered crude oil at the water-air interface formed water-in-oil emulsions, an intermediate product of crude oil that often forms in offshore waters following oil spills [13]. Floating oily particulate matter (also called ‘chocolate mousse’, ‘oil flakes’ or ‘oil pancakes’[14]) often forms oil aggregates that, after losing their buoyancy, sink out of the surface ocean and accelerate the vertical flux of oil towards the seafloor [15], [16]. Despite the fact that oil aggregates are important intermediate products of spilled crude oil, the dynamics of oil-degrading microorganisms associated with oil aggregates are, to the best of our knowledge, unexplored. Here we investigate bacterial numbers and activities of extracellular enzymes indicative for oil-degrading bacteria in surface seawaters collected near the Deepwater Horizon oil spill site in which oil aggregates were formed. The focus on enzymatic activity stems from the fact that heterotrophic microorganisms must generate extracellular enzymes to cleave their target substrates outside the cell to sizes small enough for uptake [17]. Rates and activities of microbial extracellular enzymes are therefore a good measure for the initial step of carbon cycling in natural microbial communities [18], and are used here as indicators of heterotrophic microbial activities in oil-contaminated seawater.

Microbial activities during aggregate formation and aging were studied in a model system of rotating glass bottles (roller bottles) following the experimental procedure of Shanks and Edmondson [19]. Aggregates formed in roller bottles resemble natural marine aggregates in terms of size and composition and are thus suitable to study associated microbial processes [15]. Previous work on microbial community activity has demonstrated that aggregate formation has profound effects on the initial phase of organic matter degradation in terms of extracellular enzymatic activities and rates [16]. The goal of this study was to investigate the impact of aggregate formation in oil-contaminated waters on the activities of specialized oil-degrading microorganisms as well as on the native seawater microbial communities. To differentiate these communities, we investigated enzymatic activity in seawater containing a natural seawater microbial community that was then contaminated with an oil slick, in essence an oil-associated microbial inoculum. We also measured enzymatic activities in oil-contaminated seawater incubations where the seawater had been filtered and autoclaved prior to the addition of the oil, such that the only active organisms would be those associated with the added oil. Our objective was to test the hypothesis that the formation of oil aggregates enhances enzymatic activities of oil-degrading bacteria, regulating remineralization and mobilization of oil-derived carbon to dissolved organic carbon (DOC), with consequences for the overall carbon cycle in oil-contaminated waters.

## Materials and Methods

### Water sampling

Water samples were collected from May 05 to May 08, 2010, by bucket from the RV *Pelican* (total water volume collected for this study: 9 L) during the first oil spill response cruise [3], [20]. Uncontaminated surface seawater that had no visible oil was collected 20 nautical miles east of the spill site (28.741°N, 88.001°W). Water salinity was 32 PSU at a temperature of 25°C. An oil slick floating at the sea surface (total volume of the oil-seawater mixture collected for this study: 400 mL) was sampled less than 1 nautical mile east of the spill site (28.736°N, 88.372°W). All water samples were stored in 500-ml glass jars for 4 weeks at 4°C until beginning the incubation experiments at the University of North Carolina.

### Roller table incubation

Duplicate 1-L Pyrex© glass bottles (total volume: 1150 mL) were filled to the 1-L mark with either uncontaminated seawater (SW1 and SW2) or seawater mixed with 12 ml of oil slick (SW+oil1 and SW+oil2) yielding an approximate oil slick content of 1% (v/v). In addition, one 1-L glass bottle was filled with 0.1-µm filtered and autoclaved seawater (control SW), and one bottle was filled with control seawater mixed with 12 ml of oil slick (control SW+oil). After keeping all bottles at 25°C in the dark for 60 hours to let microorganisms adjust to the temperature change, the bottles were incubated at 25°C on a roller table and were rotated at 3.5 rpm for 21 days in the dark. Bottle rotation introduced small-scale turbulence at the headspace-water interface that enabled us to study biogeochemical processes in the water under mildly turbulent conditions.

Roller bottles were separately sampled at 7 time points during the incubation (0, 2, 7, 10, 14, 16, and 21 days). At each time point, bottles were placed upright on the bench top to allow aggregates to settle to the bottom. Photos of the bottles were taken to document aggregate formation, and water samples were withdrawn from the middle of the bottles using a 10-mL glass pipette. This water contained no visible aggregates, and is hereafter referred to as ambient water. Subsamples of ambient water for cell counts and enzyme activity measurements were processed immediately after sampling. For dissolved organic matter analysis (DOC and CDOM; see below), 15 mL of ambient water were filtered through 0.2-µm surfactant free cellulose acetate (SFCA) syringe filters and stored at −20°C in precombusted scintillation vials until analysis.

At the end of the roller table incubation (day 21) all visible aggregates were siphoned from the bottom of the bottles and transferred into separate 50-mL centrifuge tubes. The tubes containing aggregates and ambient water were stored at 4°C overnight to allow aggregates to settle to the bottom. After removing the supernatant water, the tubes were weighed to determine aggregate wet weight. The tubes were then filled with control (autoclaved) seawater to the 40-mL mark to yield sufficient volume for the suite of analyses described below. We accounted for the different dilution factors for the calculation of oil aggregate hydrolysis rates (reported per mL aggregate per hour) as well as oil aggregate cell numbers (reported per mL aggregate).

### Enzyme activity measurements

Potential hydrolysis rates were measured using 4-methylumbelliferone (MUF) and 4-methylcoumarinyl-7-amide (MCA) labeled substrate analogs according to Hoppe [21]. Lipase activity (4-MUF-butyrate; final concentration: 20 µM) was measured to monitor enzyme activities of bacteria in the oil-degrading microbial cascade. We concurrently measured activities of alkaline phosphatase (4-MUF-phosphate; 10 µM), β-glucosidase (4-MUF-β-D-glucopyranoside; 1000 µM), and peptidase (L-leucine-MCA hydrochloride; 1200 µM; all substrates from Sigma-Aldrich). All substrates were added at saturation levels, as determined in preliminary experiments. Changes in fluorescence over time, corrected for the readings in control SW that were always lower than those in the live water, were used to calculate hydrolysis rates on a volume and on a cell-specific basis. The latter was used to compare aggregate-associated and ambient water hydrolysis rates at day 21. For a detailed description of enzyme activity measurements, see supplemental methods (Methods S1).

### Microbial cell abundance

Microbial cell abundance was determined according to Porter and Feig [22] and Velji and Albright [23] using 4′, 6-diamidino-2-phenylindole (DAPI) as a stain. For further information, see supplemental methods (Methods S1).

### Dissolved organic carbon (DOC and CDOM)

Filtered and frozen samples from each time point were thawed and duplicate samples were withdrawn and acidified with phosphoric acid (50% v/v) to measure DOC concentrations by high temperature catalytic oxidation using a Shimadzu TOC-5000. Measurements of a single sample were repeated three times, and average values ± standard deviations were calculated from n = 6 measurements. CDOM absorbance was measured in un-acidified samples on a Varian 300 UV spectrophotometer using MilliQ water as a reference, while CDOM fluorescence was measured on a Varian Eclipse fluorometer, also using MilliQ water as a reference [24]. For more details on CDOM analysis, see supplemental methods (Methods S1).

### Statistical analysis

Correlation coefficients as well as differences between two average values given as their statistical mean ± standard deviation were tested for their significance using the Students t-test. Analysis of variance (one-way ANOVA) was used for comparing average values of more than two groups of data. If ANOVA was significant, post hoc pairwise comparisons of means were performed using the Bonferroni-Holmes test of variability. All statistical analysis was performed in Excel® using the data analysis toolpack as well as Daniel's XL toolbox (both open source add-ins).

## Results

### Formation of oil aggregates in roller bottles

**Figure 1:**
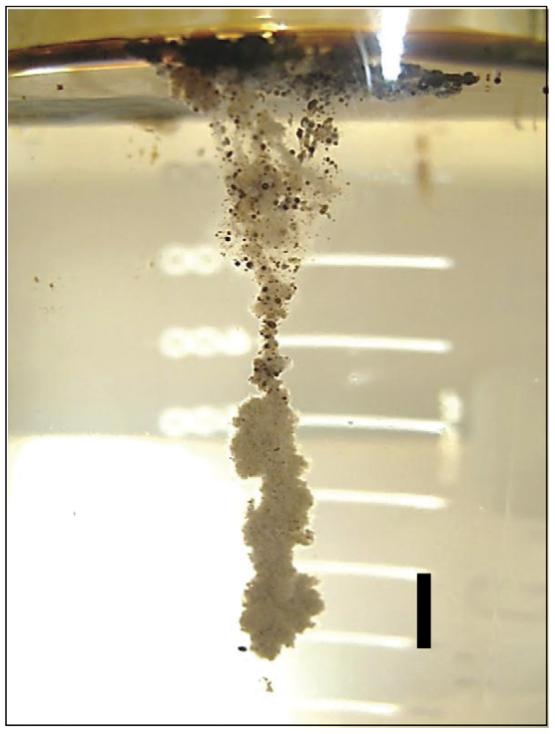
Photo of an oil aggregate formed in one of the roller bottles. Oil aggregate attached to surface water oil slick through sticky oil gels. Photo was taken at the end of the 21-day roller table incubation in one of the roller bottles containing seawater and oil (SW+oil1). Scale bar is approximately 10 mm.

Roller table incubation of uncontaminated seawater collected near the Deepwater Horizon oil spill site with surface oil sampled in the same area (hereafter referred to as SW+oil1 and SW+oil2 bottles; see Material and methods for roller bottles set-up) led to rapid formation of aggregates (hereafter referred to as oil aggregates) within one day (see Fig. S1 for close-up photos of oil aggregates). Oil aggregates in both SW+oil bottles clumped together after 7 days, forming a single aggregate up to 30 mm in diameter, with visibly incorporated oil droplets. Oil aggregate formation in control SW+oil (seawater that had been filtered and autoclaved before the oil was added) was first observed at day 10 after the appearance of gelatinous networks of particulate matter with incorporated oil droplets (hereafter referred to as oil gels). Oil gels appeared to be very sticky surfaces onto which oil aggregates attached upon collision (Fig. 1).

In contrast to oil-amended bottles, aggregate formation in roller bottles with seawater not amended with oil (SW bottles) was delayed and reduced in scale (aggregates that were much more transparent than oil aggregates first appeared after 3 days; Fig. S1E), and aggregates were less abundant (2 to 3 per bottle). These aggregates did not change in size and number throughout the 21 days of incubation. No aggregates formed in the control SW bottle containing filtered and sterilized seawater.

Wet weights of oil aggregates in SW+oil1, SW+oil2, and control SW+oil bottles after 21 days were 5.5 g, 7.1 g, and 9.8 g, respectively. Assuming a final bottle water volume of 900 ml at day 21 (weight≈924.3 g), oil aggregates occupied approximately 0.6% (SW+oil1), 0.8% (SW+oil2), and 1.1% (control SW+oil) of the total roller bottle volume. SW1 and SW2 aggregates were 0.19 g and 0.17 g, respectively, and thus 0.02% of total roller bottle volume.

### Microbial cell abundance in ambient waters

**Figure 2:**
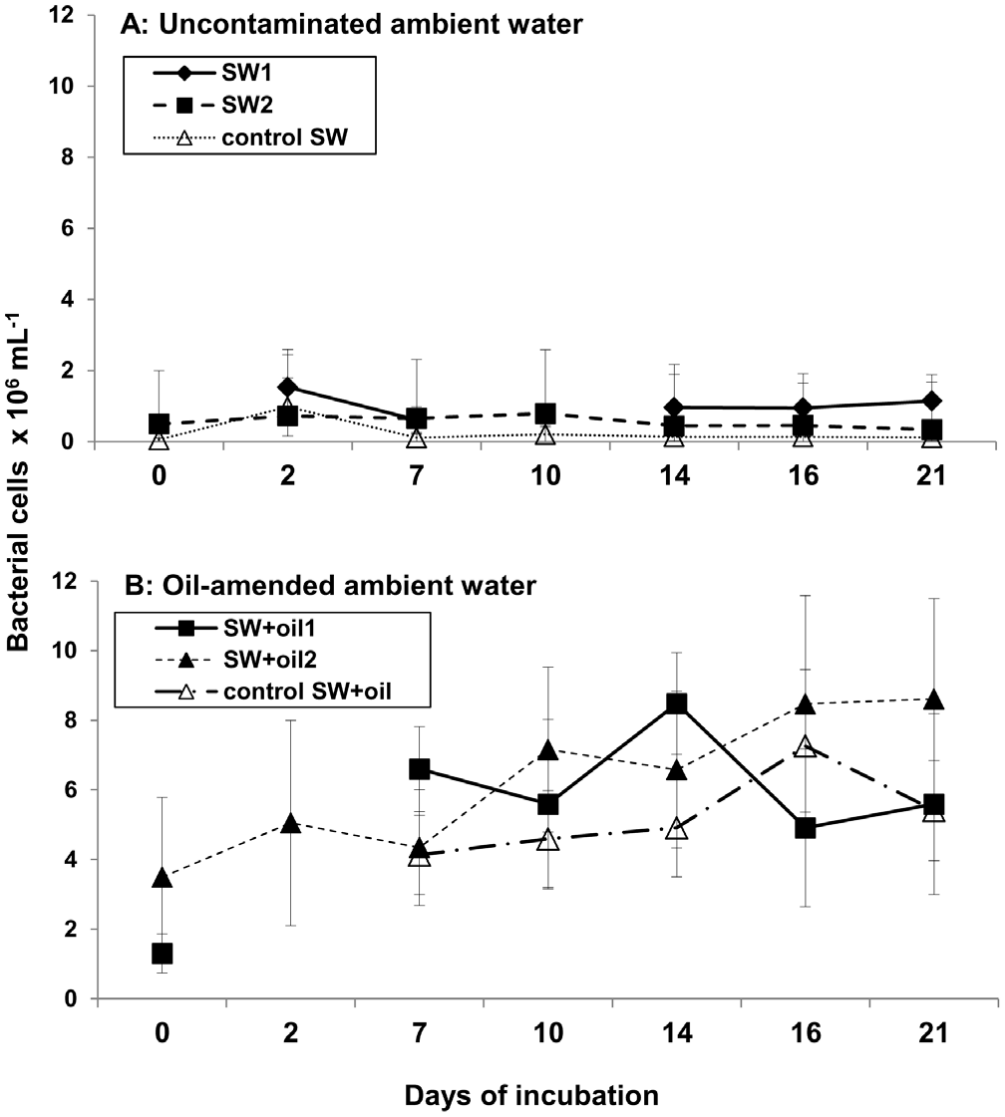
Microbial cell numbers in roller bottle ambient waters. Average microbial cell numbers in (A) uncontaminated and (B) oil-amended ambient water. Error bars represent standard deviations of 10 counting fields. Note that there are no cell counts available for control SW at day 2, SW1 at day 0 and day 10, SW+oil1 at day 2, and control SW+oil at day 0 and day 2.

The microbial cell counts documented the impact of oil amendments on the abundance of prokaryotic cells in surface seawater during the roller table incubations, compared to uncontaminated seawater. Uncontaminated ambient water (SW2) had 0.5±0.4×10^6^ cells mL^−1^ at day 0 (Fig. 2A); this number was lower (*p*<0.05) but the same order of magnitude as the cell abundance of uncontaminated water fixed shortly after sampling (0.8±0.2×10^6^ mL^−1^), indicating that storage time and conditions from the time of sampling until the beginning of the experiment had little influence on cell numbers in uncontaminated water (note that a fixed sample of the oil slick was not available). Initial SW2 cell numbers were also lower than the cell numbers from SW+oil1 (1.3±0.6×10^6^ mL^−1^) and SW+oil2 (3.5±2.3×10^6^ mL^−1^) at day 0 of the experiment (*p*<0.01), suggesting that bacterial cells were introduced into oil-amended bottles along with the oil sample.

Throughout the incubation, cell numbers in uncontaminated bottles remained low and were either indistinguishable from one another (control SW, *p* = 0.2; SW1, *p* = 0.2), or decreased towards the end of the incubation (SW2, *p*<0.05). In contrast, SW+oil1 cell numbers increased after the start of the incubation (all time points were significantly higher than day 0, *p*<0.001), peaking at day 14 (8.5±1.5×10^6^ mL^−1^; Fig. 2B). SW+oil2 cell numbers were significantly higher at day 10, 16, and 21 compared with day 0 (*p*<0.001), and control SW+oil cells showed significantly higher numbers at day 16 (7.3±2.2×10^6^ mL^−1^) than day 7 (4.1±1.1×10^6^ mL^−1^, *p*<0.001; note that no cell counts are available for days 0 and 2 due to high autofluorescence of the samples).

### Microbial cell abundance in oil aggregates

Aggregate-associated microbial cells accounted for high proportions of the total cell counts in the oil-amended incubations. Average cell numbers in oil aggregates at day 21 were 11±0.01×10^8^ (mL aggregate)^−1^ in SW+oil1 as well as 4±0.01×10^8^ (mL aggregate)^−1^ in SW+oil2 and control SW+oil. SW1 and SW2 aggregates had 16.7±0.04×10^8^ cells (mL aggregate)^−1^ and 28.2±0.01×10^8^ cells (mL aggregate)^−1^, respectively (data not shown). Corrected for their approximate volume in each of the roller bottles (e.g. SW+oil1 aggregates: 0.6% of 900 ml bottle water≈5.4 ml oil aggregates), total aggregate-associated cell numbers in oil-amended bottles were 60.7±0.05×10^9^ (SW+oil1), 28.4±0.05×10^9^ (SW+oil2), and 39.3±0.05×10^9^ (control SW+oil; Fig. S2). Uncontaminated bottles had fewer cells associated with aggregates compared to oil-amended bottles (*p*<0.001), with total aggregate-associated cell numbers at 3.2±0.01×10^9^ (SW1) and 4.7±0.02×10^9^ (SW2). Total aggregate-associated cell numbers compared with total cell numbers in ambient waters at day 21 (i.e. cell number per mL at day 21 (Fig. 2) multiplied by the approximate volume of the ambient water at day 21) revealed that on average 55% and 45% of cells in SW+oil1 were associated with oil aggregates and suspended in ambient waters, respectively (total numbers not significantly different from one another, *p* = 0.08; Fig. S2). In SW+oil2, 27% (*p*<0.001) of the cells were aggregate-associated; 45% (*p* = 0.08) of cells in control SW+oil were attached to aggregates. SW1 had 24% of cells attached to aggregates (*p*<0.01) while 61% of SW2 cells were aggregate-associated (*p*<0.05).

### Enzyme activities in ambient waters

**Figure 3:**
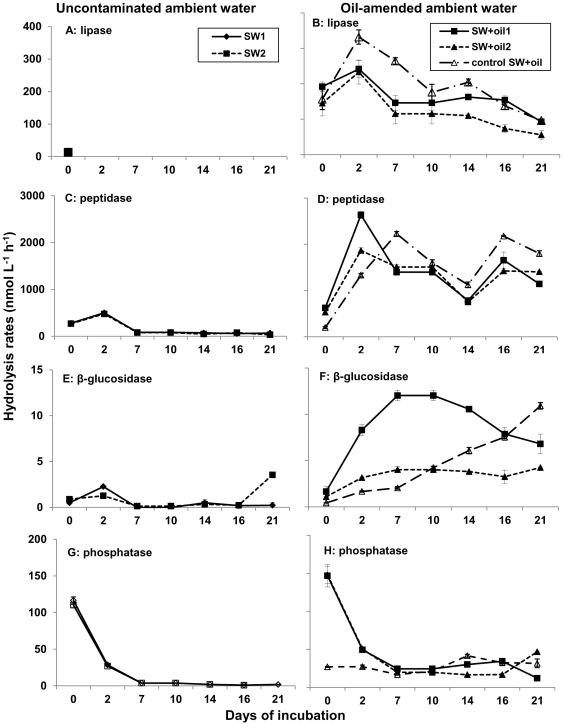
Enzyme activities in roller bottle ambient waters. Average potential hydrolysis rates (n = 3 ± standard deviation) in uncontaminated (A, C, E, G) and oil-amended (B, D, F, H) ambient waters. Note the different scales on the y-axis.

Lipase, peptidase, and β-glucosidase activities showed different magnitudes and time course patterns in uncontaminated and in oil-amended ambient waters (Fig. 3A–F). Lipase activities in oil-amended ambient waters were high throughout the incubation, ranging between 55.6±12.3 nmol L^−1^ h^−1^ in SW+oil2 at day 21 and 332±20.1 nmol L^−1^ h^−1^ in control SW+oil at day 2 (Fig. 3B). In contrast, lipase activity in uncontaminated bottles was measurable only at day 0 (SW1: 14.9±11.9 nmol L^−1^ h^−1^ and SW2: 13.5±0.13 nmol L^−1^ h^−1^; Fig. 3A), and on average one order of magnitude lower than in oil-amended bottles at day 0 (*p*<0.001). Throughout the incubation, lipase activities in oil-amended ambient waters showed an overall decrease (SW+oil1 and SW+oil2: *r* = −0.8; *p*<0.05; control SW+oil: *r* = −0.6; *p* = 0.13; all *n* = 7). Despite similar time courses, control SW+oil activities were significantly higher than SW+oil bottles at day 2 (*p*<0.01), day 7 and day 14 (both *p*<0.001), and high but indistinguishable from SW+oil1 at day 16 (*p* = 0.13) and 21 (*p* = 0.26).

Initial peptidase activities in oil-amended and uncontaminated bottles were similar, ranging between 199±19.8 nmol L^−1^ h^−1^ (control SW+oil) and 625±27.5 nmol L^−1^ h^−1^ (SW+oil1; Fig. 3C, D); activities at day 0 decreased in the following order: SW+oil1>SW+oil2 >SW1 and SW2>control SW+oil (*p*<0.001). Throughout the incubation, peptidase activities in uncontaminated seawater were considerably lower compared to oil-amended bottles, where peptidase activities increased on average one order of magnitude at day 2 and remained high throughout the rest of the incubation. Control SW+oil activities were highest at day 7, day 14, and day 21 (all *p*<0.001), and high but indistinguishable from SW+oil2 at day 10 (*p* = 0.23) and SW+oil1 at day 16 (*p* = 0.028).

For β-glucosidase, initial activities in all five bottles were indistinguishable from one another (*p* = 0.26), ranging between 0.5±0.02 nmol L^−1^ h^−1^ in SW1 and 1.7±0.6 nmol L^−1^ h^−1^ in SW+oil2 (Fig. 3E, F). Oil-amended activities increased sharply with time (SW+oil1: *r* = 0.27; *p* = 0.55; SW+oil2: *r* = 0.67; *p* = 0.09; control SW+oil: *r* = 0.98; *p*<0.05), but uncontaminated activities remained low and showed no distinct pattern throughout the incubation.

Patterns of phosphatase activities were similar among all live incubations, with a sharp activity drop between day 0 and day 2 (Fig. 3G, H). In this time period, control SW+oil activities were lowest (*p*<0.001). After day 2 phosphatase activities remained low, showing no distinct patterns for the rest of the incubation.

### Enzyme activities in oil aggregates

**Figure 4:**
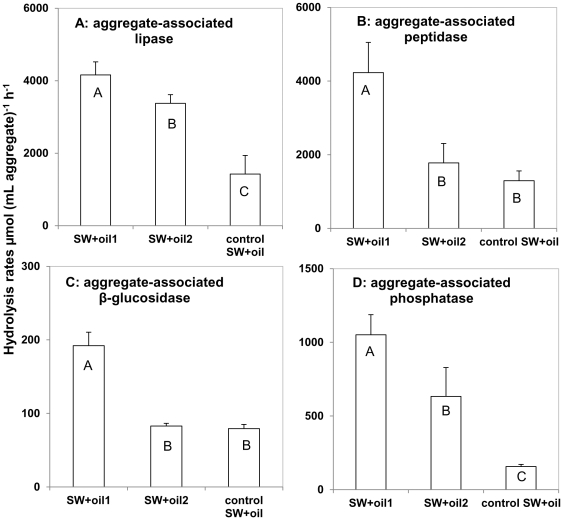
Aggregate-associated enzyme activities at the end of the incubation. Average potential hydrolysis rates (n = 3 ± standard deviation) in oil aggregates after the 21-day roller table incubation. Letters indicate results from one-way ANOVA followed by the Bonferroni-Holmes test. Rates with the same letter are statistically indistinguishable. Note the different scales on the y-axis.

Highest aggregate-associated enzyme activities at day 21 for all four substrates, on a volume basis, were found in SW+oil1 (Fig. 4; note that all of the enzymatic activities in SW aggregates remained below detection limit), decreasing in the following order: peptidase (4231±818 µmol [mL aggregate]^−1^ h^−1^) and lipase (4161±359 µmol [mL aggregate]^−1^ h^−1^) > phosphatase (1051±137 µmol [mL aggregate]^−1^ h^−1^) > β-glucosidase (192±18 µmol [mL aggregate]^−1^ ^−1^ h^−1^; *p*<0.001). Control SW+oil aggregates had the lowest activities for lipase and phosphatase (both *p*<0.001) while peptidase and β-glucosidase activities were indistinguishable from one another in SW+oil2 (*p* = 0.23 and *p* = 0.45, respectively). On a cell-specific basis, aggregate-associated enzyme activities were lower compared with ambient waters at day 21 (Fig. S3). This pattern was most pronounced for peptidase activity, where cell-specific activity in oil-aggregates was up to three orders of magnitude lower than in oil-amended ambient waters (Fig. S3B).

### Dissolved organic matter (CDOM and DOC)

**Figure 5:**
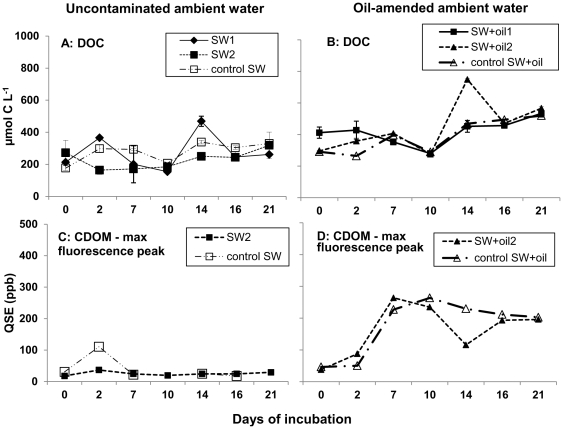
DOC and CDOM in roller bottle ambient water. Dissolved organic carbon (DOC) concentration (A and B; n = 6 ± standard deviation) and colored dissolved organic matter (CDOM) fluorescence (maximum fluorescence peak; C, D) during the 21-day roller table incubation. Note that CDOM fluorescence was not measured in SW1 and SW+oil1. There are no data available on CDOM fluorescence for control SW at day 21.

The concentration of DOC in the roller bottles was monitored in order to detect oil-associated DOC changes over time. Initial DOC concentrations in uncontaminated bottles ranged between 177±15.3 µmol C L^−1^ (control SW) and 213±2 µmol C L^−1^ (SW2; Fig. 5A). Concentrations in SW+oil2 (295±5 µmol C L^−1^) and control SW+oil (291±2 µmol C L^−1^) were higher but indistinguishable from SW2; overall highest DOC concentration at day 0 was found in SW+oil1 at 412±35.4 µmol C L^−1^ (*p*<0.001; Fig. 5B). Throughout the incubation, DOC increased on average twofold in all three oil-amended bottles, resulting in significantly higher concentrations at day 21 compared with uncontaminated bottles (*p*<0.001).

**Figure 6:**
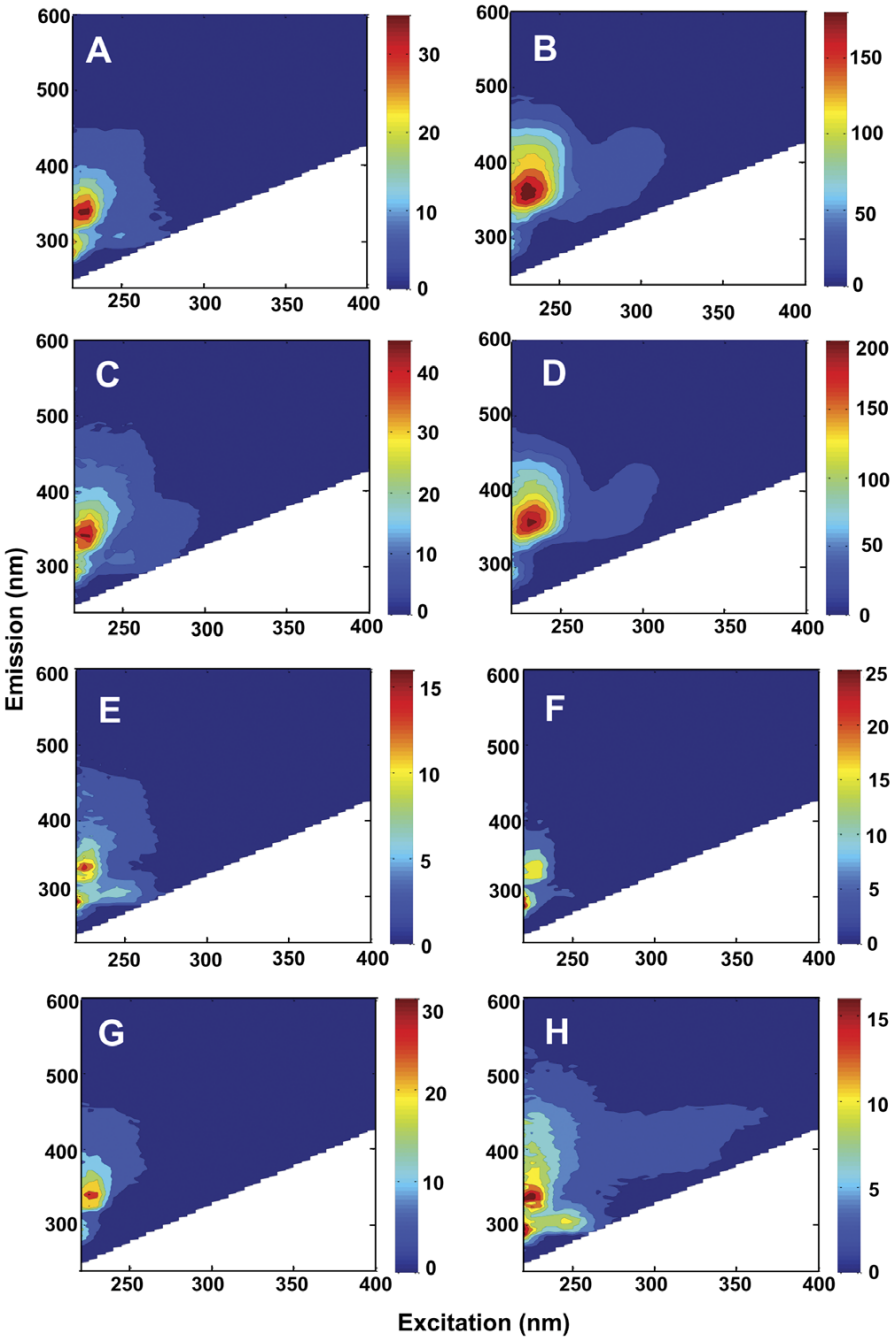
Excitation-emission matrices (EEMs) for day 0 and day 21; note difference in scale among panels. (A) SW+oil2, day 0; (B) SW+oil2, day 21; (C) control SW+oil, day 0; (D) control SW+oil, day 21; (E) SW2, day 0; (F) SW2, day 21; (G) control SW, day 0; (H) control SW, day 16 (there are no data available for day 21). The fluorescence intensities in oil-amended bottles (panels B and D) at day 21 were one order of magnitude higher than for all other samples.

Fluorescence properties of colored dissolved organic matter (CDOM), as monitored by the maximum fluorescence peak, revealed only minor changes in uncontaminated bottles over time (Fig. 5C). In contrast, SW+oil2 and control SW+oil bottles showed a sharp increase in CDOM fluorescence within the first week of the incubation, with fluorescence signals up to two orders of magnitude higher than in SW bottles (note that SW1 and SW+oil1 were not analyzed for CDOM fluorescence). In addition, excitation-emission matrices in oil-amended roller bottles (SW+oil2 and control SW+oil) showed an up to 10-fold increase of fluorescence and a slight “red-shift” in the peak emission fluorescence from 340 nm to 360 nm during the course of the incubation (Fig. 6 A–D). In contrast, SW2 and control SW showed only a modest increase in fluorescence intensity and no red-shift in emission peak position (Fig. 6 E–H).

## Discussion

We used rotating bottles to simulate the effects of contamination of surface seawater with an oil slick from the Deepwater Horizon spill site. Observation of oil aggregate formation and dynamics over a 21 day time course, coupled with measurements of a suite of potential extracellular enzyme activities, demonstrated that oil contamination led to increased microbial activities compared with uncontaminated surface seawater from the spill site. High rates of bacterial oil degradation from this spill have been reported from deep water microbial communities [1], [5], [7], [9]. To the best of our knowledge, this is the first study reporting the effects of the Deep Water Horizon oil spill on hydrolytic enzyme activities and DOC dynamics of oil-impacted surface water bacterial communities.

The patterns and rates of enzymatic activities as well as dynamics of dissolved organic matter in SW+oil (natural microbial community plus microbial inoculum associated with the oil slick) and control SW+oil bottles (microbial inoculum associated with the oil slick only) suggest that activities of microbes associated with the oil slick were high throughout the 21 days, and stimulated the metabolism of the natural seawater microbial community. The presence of the two microbial communities enhanced overall bacterial growth, as indicated by increasing cell numbers over time in oil-amended bottles (Fig. 2), and biogeochemical processes, including dynamics of oil aggregates within oil-amended roller bottles. In particular, enhanced bacterial breakdown of petroleum hydrocarbons, indicated by elevated lipase activities in oil-amended ambient waters (Fig. 3B), may have led to an increase in polar components within roller bottles during the initial phase of the incubation. Polar components such as asphaltenes and resins [11] often form stable water-in-oil emulsions with a reddish to brown appearance in waters affected by oil spills ([25] and references therein). In our experiments, asphaltenes and resins may have served as coagulation kernels, stimulating the rapid formation of oil aggregates (Figs. 1, S1).

Lipase activity patterns are consistent with the hypothesis that oil-degrading bacteria may have initially degraded the easily metabolized, soluble components of oil, and then have progressively colonized the oil aggregates. The time lag of oil-aggregate formation in control SW+oil compared to SW+oil bottles could then explain higher ambient water lipase activities in the former during the first week of the incubation (Fig. 3B).

Increasing bacterial colonization of oil aggregates throughout the incubation resulted in high bacterial biomass (Fig. S2) and lipase activity in oil aggregates at the end of the incubation (Fig. 4A; Fig. S3A). Enhanced bacterial degradation of oil in aggregates likely increased the carbon flux into the ambient water, resulting in a two fold increase of DOC concentrations in oil-amended compared to uncontaminated roller bottles by the end of the incubation (Fig. 5). DOC concentrations in uncontaminated seawater are consistent with previously reported DOC concentrations measured in uncontaminated surface waters on the Louisiana shelf near our sampling site [26].

Further evidence for an enhanced carbon flux from oil aggregates into ambient waters driven by bacterial oil degradation comes from patterns of CDOM fluorescence. In particular, we found approximately 10-fold higher fluorescence intensities as well as a “red-shifted” fluorescence signal in oil-amended bottles relative to uncontaminated bottles at the end of the incubation (Fig. 6A–D), suggesting an increase over time of aromatic hydrocarbons in oil-amended bottles [27], [28]. Considering that DOC and CDOM patterns showed only minor changes in uncontaminated bottles, we conclude that bacterial processing in aggregates drove the flux of oil-derived carbon into the DOC pool, affecting the quality and quantity of the DOC pool in ambient waters.

This increasing flux of mainly oil-derived DOC into ambient waters stimulated bacterial activities not directly associated with oil degradation, indicated by increasing microbial biomass (Fig. 2) as well as high levels of peptidase and β-glucosidase activities (Fig. 3D, F). Peptidase activities, on a volume basis, were up to a factor of 70 higher than rates previously measured on the Louisiana Shelf near our sampling site [29]. Comparison of cell-specific peptidase activities at day 21 also revealed up to three orders of magnitude higher hydrolysis rates in oil-amended compared to uncontaminated ambient waters (Fig. S3B). Activities of β-glucosidase, on a volume and a cell-specific basis, were also elevated in oil-amended ambient waters compared to uncontaminated waters (Figs. 3C; S3C), and similar to hydrolysis rates previously measured in organic matter-rich, estuarine Gulf of Mexico waters [30].

High rates of peptide as well as carbohydrate hydrolysis in roller bottles may have been stimulated by enhanced production and release of metabolites from oil degrading bacteria, in particular extracellular polymeric substances (EPS) that emulsify crude oil and increase bioavailability for microbial processing [31]. Gutierrez et al. [32] demonstrated that oil-emulsifying EPS extracted from *Halomonas* species consist mainly of glycoproteins with high amounts of polysaccharides, compounds that could enhance activities of carbohydrate- and peptide-degrading enzymes. Moreover, laboratory incubations suggest that members of the genus *Cycloclasticus*, a known aromatic hydrocarbon degrader in the ocean [33], may only grow on crude oil emulsified by microbial EPS [34]. Enhanced production of EPS by oil degrading bacteria in our oil-amended bottles may have led to the formation of oil gels (Figs. 1, S1), increasing their stickiness and thus affecting aggregate dynamics. Abundant EPS in aggregates at the end of the incubation would then also explain high levels of aggregate-associated peptidase and β-glucosidase activities (Fig. 4).

Our investigation of the effects of oil contamination on the dynamics and activities of natural microbial communities may be a good model for processes that drove the vertical transport of oil-derived particulate matter through the water column in the aftermath of the Deepwater Horizon oil spill. In particular, floating oil aggregates, similar in size and appearance to the ones we observed in the roller bottles, were observed in surface waters near the spill site two weeks after the onset of the spill (Fig. S4). Formation of oil aggregates subsequent to oil spills in the Gulf of Mexico has been reported previously. Following the Ixtoc I oil spill in 1979, Patton et al. [35] observed oil aggregates but did not study aggregate-associated microbial processes, although they argued that microorganisms that colonized oil aggregates played only a minor role in oil aggregate dynamics. Our data suggest, in contrast, that oil aggregates may be hotspots for oil degrading microbial activities.

During the Ixtoc I spill, oil aggregates sank out of the surface ocean, causing a major fraction of the spilled oil to settle to the seafloor [36]. In the case of the Deepwater Horizon spill, visual observations as well as chemical analysis of sediments in the vicinity of the wellhead also indicated local oil sedimentation and contamination of deep sea sediments [1], [37]. Sedimentation of oily particulate matter may have been accelerated by the formation and sinking of oil aggregates, similar to the processes observed during the Ixtoc I spill. In fact, sinking oil aggregates near the wellhead were observed throughout the water column in the second half of May, 2010 (Arne-R. Diercks, pers. comm.). With an estimated sinking velocity of 75 to 120 m d^−1^ (calculated using the size range of oil aggregates formed in roller bottles and the equation from [38]), sinking oil aggregates would have reached the seafloor, assuming a water depth of 1500 m, in about one week. During sinking, oil aggregates likely transferred oil- and EPS-derived DOC into the water column, thus mediating the transport of particulate and dissolved carbon derived from bacterial oil degradation, as well as oil-degrading microbes themselves, from the surface into the deep sea.

## Supporting Information

Figure S1 **Aggregate formation in roller bottles.** Aggregates formed within oil-amended roller bottles (SW+oil; A, B; control SW+oil; C, D), and uncontaminated bottles (E) at different times during the incubation. Scale bar is approximately 5 mm.
(TIF)

Figure S2 **Total bacterial numbers at day 21.** Total cell numbers normalized to the volume of ambient water and oil aggregates per bottle (see text for details). Error bars represent standard deviations of 10 counting fields; note that the standard deviations from average aggregate-associated cell numbers were low, ranging between 0.1% and 0.4%. Letters indicate results from one-way ANOVA followed by the Bonferroni-Holmes test (capital letters: ambient water; small letters: aggregates). Cell numbers with the same letter are statistically indistinguishable. Asterisks indicate significant differences among ambient water and aggregate-associated cell numbers per bottle based on Student's t-test at *p<0.05, **p<0.01, and ***p<0.001.
(TIF)

Figure S3 **Cell-specific enzyme activities in ambient waters and oil aggregates at day 21.** Hydrolysis rates (n = 3 ± standard deviation) on a cell-specific basis. Letters indicate results from one-way ANOVA followed by the Bonferroni-Holmes test. Rates with the same letter are statistically indistinguishable. Data labels are average hydrolysis rates. Note the different scales on the y-axis; n.d. means not detectable.
(TIF)

Figure S4 **Floating oil aggregates in surface water near the spill site.** Photo taken by Arne-R. Diercks (NIUST) on May 11, 2010, onboard the R/V *Pelican*.
(TIF)

Methods S1 **Detailed description of enzyme activity, cell counts and CDOM measurements.**
(PDF)

## References

[1] Atlas RM, Hazen TC (2011). Oil biodegradation and bioremediation: A tale of the two worst spills in US history.. Environ Sci Technol.

[2] McNutt M, Camilli R, Guthrie G, Hsieh P, Labson V (2011). Assessment of flow rate estimates for the Deepwater Horizon/Macondo well oil spill..

[3] Diercks AR, Highsmith RC, Asper VL, Joung D, Zhou Z (2010). Characterization of subsurface polycyclic aromatic hydrocarbons at the Deepwater Horizon site.. Geophys Res Lett.

[4] Reddy CM, Arey JS, Seewald JS, Sylva SP, Lemkau KL (2011). Composition and fate of gas and oil released to the water column during the Deepwater Horizon oil spill.. Proc Natl Acad Sci U S A.

[5] Camilli R, Reddy CM, Yoerger DR, Van Mooy BAS, Jakuba MV (2010). Tracking hydrocarbon plume transport and biodegradation at Deepwater Horizon.. Science.

[6] Hazen TC, Dubinsky EA, DeSantis TZ, Andersen GL, Piceno YM (2010). Deep-sea oil plume enriches indigenous oil-degrading bacteria.. Science.

[7] Kessler JD, Valentine DL, Redmond MC, Du M, Chan EW (2011). A persistent oxygen anomaly reveals the fate of spilled methane in the deep Gulf of Mexico.. Science.

[8] Redmond MC, Valentine DL (2011). Natural gas and temperature structured a microbial community response to the Deepwater Horizon oil spill.. Proc Natl Acad Sci U S A.

[9] Valentine DL, Kessler JD, Redmond MC, Mendes SD, Heintz MB (2010). Propane respiration jump-starts microbial response to a deep oil spill.. Science.

[10] Valentine DL, Mezić I, Maćešić S, Črnjarić-Žic N, Ivić S (2012). Dynamic autoinoculation and the microbial ecology of a deep water hydrocarbon eruption.. Proc Natl Acad Sci U S A.

[11] Head IM, Jones DM, Röling WFM (2006). Marine microorganisms make a meal of oil.. Nat Rev Microbiol.

[12] Operational Science Advisory Team (OSAT) (2010). Summary report for fate and effects of remnant oil in the beach environment.. http://www.restorethegulf.gov/sites/default/files/u316/OSAT-2%20Report%20no%20ltr.pdf.

[13] Niu H, Li Z, Lee K, Kepkay P, Mullin JV (2011). Modelling the transport of oil–mineral-aggregates (OMAs) in the marine environment and assessment of their potential risks.. Environ Model Assess.

[14] National Research Council (NRC) (2003). Oil in the sea III: Inputs, fates, and effects.

[15] Unanue MA, Azua I, Arrieta JM, Herndl GJ, Iriberri J (1998). Laboratory-made particles as a useful approach to analyze microbial processes in marine macroaggregates.. FEMS Microbiol Ecol.

[16] Ziervogel K, Steen AD, Arnosti C (2011). Changes in the spectrum and rates of extracellular enzyme activities in seawater following aggregate formation.. Biogeosciences.

[17] Weiss MS, Abele U, Weckesser J, Welte W, Schiltz E (1991). Molecular architecture and electrostatic properties of a bacterial porin.. Science.

[18] Arnosti C (2011). Microbial extracellular enzymes and the marine carbon cycle.. Ann Rev Mar Sci.

[19] Shanks AL, Edmondson EW (1989). Laboratory-made artificial marine snow: A biological model of the real thing.. Mar Biol.

[20] Diercks AR, Asper VL, Highsmith RC, Woolsey M, Lohrenz S (2010). NIUST - deep water horizon oil spill response cruise..

[21] Hoppe HG (1983). Significance of exoenzymatic activities in the ecology of brackish water - measurements by means of methylumbelliferyl-substrates.. Mar Ecol Prog Ser.

[22] Porter KG, Feig YS (1980). The use of DAPI for identifying and counting aquatic microflora.. Limnol Oceanogr.

[23] Velji MI, Albright LJ (1986). Microscopic enumeration of attached marine bacteria of seawater, marine sediment, fecal matter, and kelp blade samples following pyrophoshate and ultrasound treatments.. Can J Microbiol.

[24] Stedmon CA, Markager S, Kaas H (2000). Optical properties and signatures of chromophoric dissolved organic matter (CDOM) in Danish coastal waters.. Estuar Coast Shelf Sci.

[25] Fingas M, Fieldhouse B (2003). Studies of the formation process of water-in-oil emulsions.. Mar Pollut Bull.

[26] Woysocki JA, Bianchi TS, Powell RT, Reuss N (2006). Spatial variability in the coupling of organic carbon, nutrients, and phytoplankton pigments in surface waters and sediments of the Mississippi River plume.. Estuar Coast Shelf Sci.

[27] Coble PG (2007). Marine optical biogeochemistry: The chemistry of the ocean color.. Chem Rev.

[28] Senesi N, Miano T, Provenzano M, Burnett G (1991). Characterization, differentiation, and classification of humic substances by fluorescence spectroscopy.. Soil Sci.

[29] Ammerman JW, Glover WB (2000). Continuous underway measurement of microbial ectoenzyme activities in aquatic ecosystems.. Mar Ecol Prog Ser.

[30] Murrell MC (2003). Bacterioplankton dynamics in a subtropical estuary: Evidence for substrate limitation.. Aquat Microb Ecol.

[31] Hino S, Watanabe K, Tatkahashi N (1997). Isolation and characterization of slime-producing bacteria capable of utilizing petroleum hydrocarbons as a sole carbon source.. J Ferment Bioeng.

[32] Gutierrez T, Mulloy B, Black K, Green DH (2007). Glycoprotein emulsifiers from two marine *Halomonas* species: Chemical and physical characterization.. J Appl Microbiol.

[33] Kasei Y, Kishira H, Harayama S (2002). Bacteria belonging to the genus *Cycloclasticus* play a primary role in the degradation of aromatic hydrocarbons released in a marine environment.. Appl Environ Microbiol.

[34] Iwabuchi N, Sunairi M, Urai M, Itoh C, Anzai H (2002). Extracellular polysaccharides of *Rhodococcus rhodochrous* S-2 stimulate the degradation of aromatic components in crude oil by indigenous marine bacteria.. Appl Environ Microbiol.

[35] Patton JS, Rigler MW, Boehm PD, Fiest DL (1981). Ixtoc 1 oil spill: Flaking of surface mousse in the Gulf of Mexico.. Nature.

[36] The Federal Interagency Solution group: Oil Budget Calculator Science and Engineering team (2010). Oil budget calculator technical documentation.. http://www.noaanews.noaa.gov/stories2010/PDFs/OilBudgetCalc_Full_HQ-Print_111110.pdf.

[37] Operational Science Advisory Team (OSAT) (2010). Summary report for sub-sea and sub-surface oil and dispersant detection: Sampling and monitoring.. http://www.dep.state.fl.us/deepwaterhorizon/files2/osat_report_17dec.pdf.

[38] Alldredge AL, Gotschalk C (1988). In situ settling behavior of marine snow.. Limnol Oceanogr.

