# Supplementary figures and images for: Microbial Activities and Dissolved Organic Matter Dynamics in Oil-Contaminated Surface Seawater from the Deepwater Horizon Oil Spill Site
Source: PLoS One. 2012 Apr 11;7(4):e34816. doi: 10.1371/journal.pone.0034816 (PMC3324544; doi:10.1371/journal.pone.0034816)

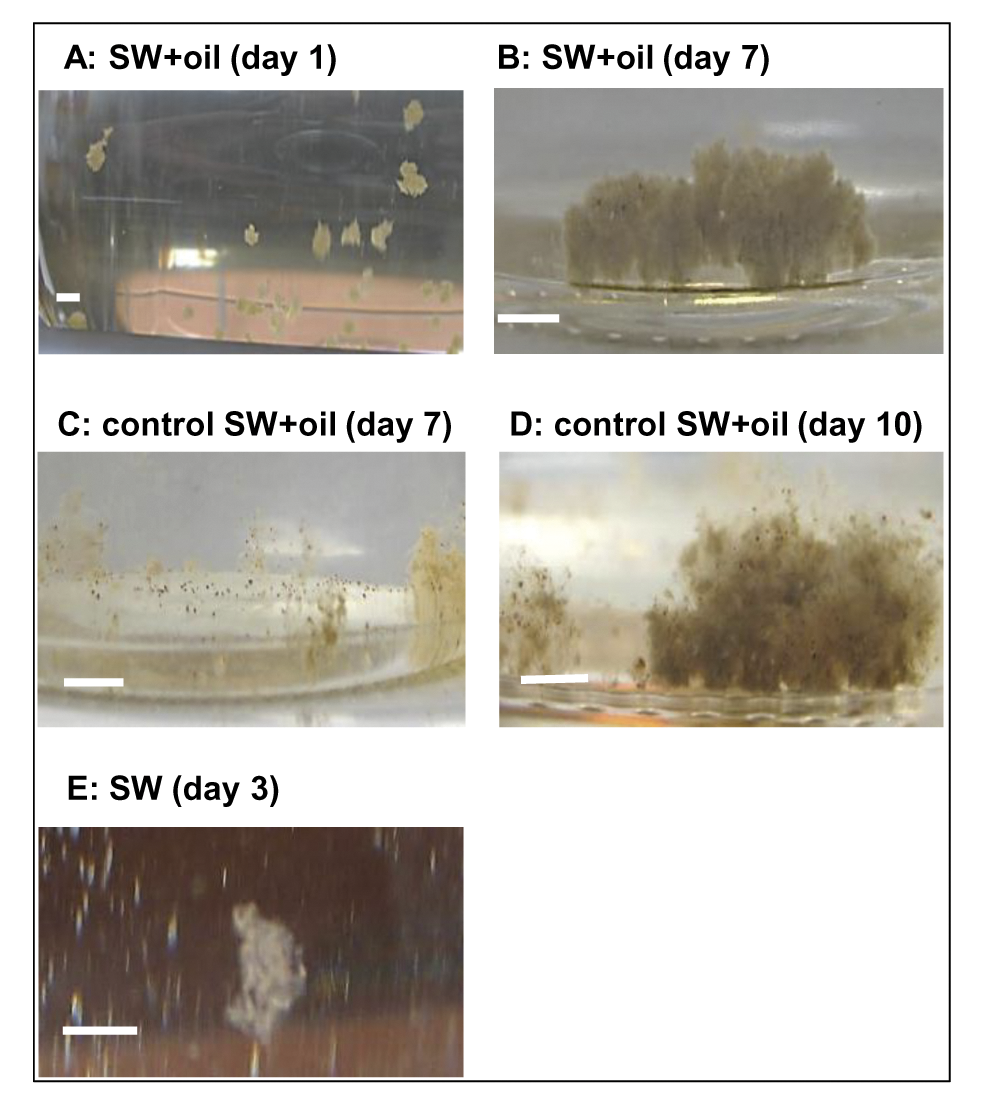

Supplement: Figure S1 — Aggregate formation in roller bottles. Aggregates formed within oil-amended roller bottles (SW+oil; A, B; control SW+oil; C, D), and uncontaminated bottles (E) at different times during the incubation. Scale bar is approximately 5 mm. (TIF) [file pone.0034816.s001.tif]

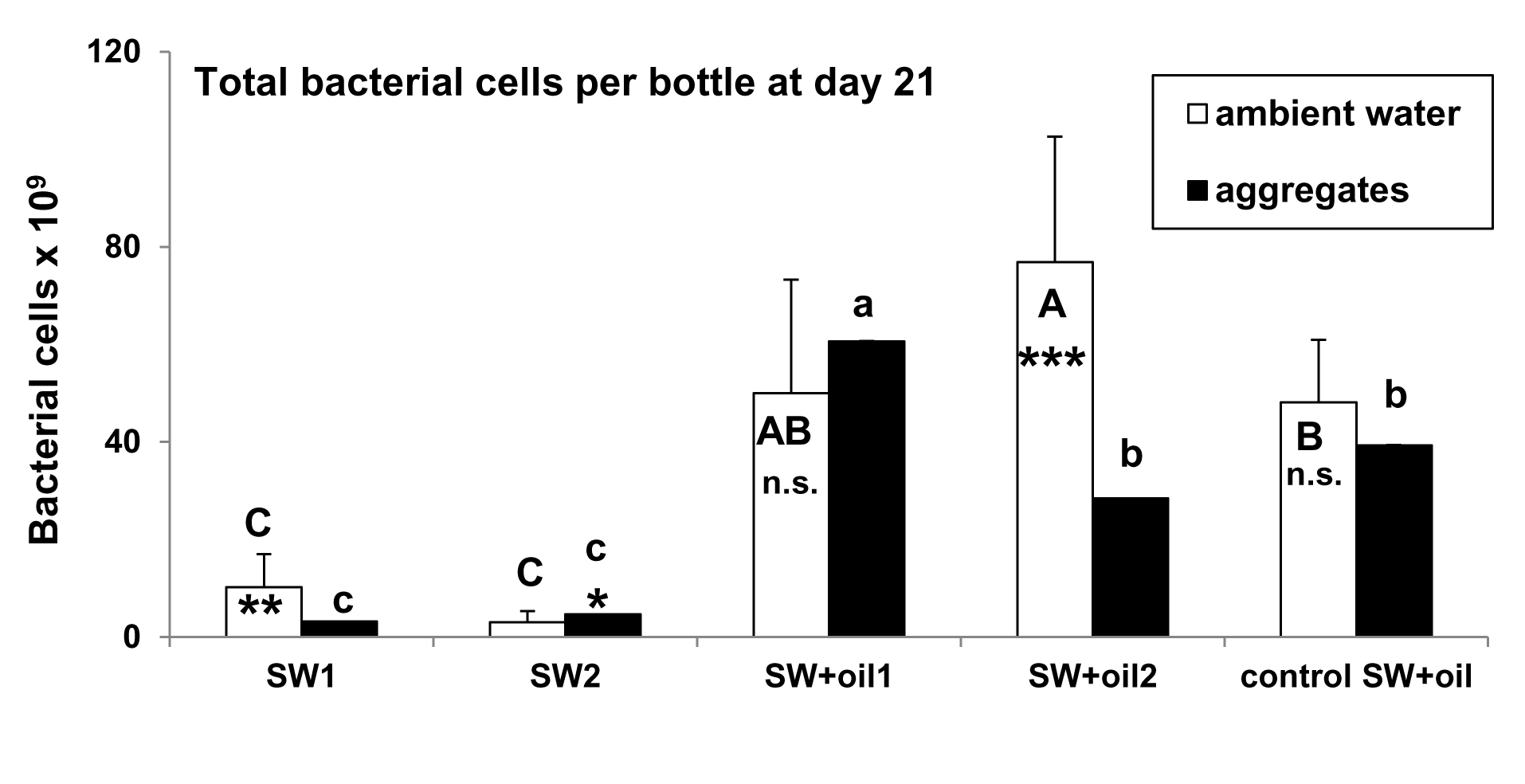

Supplement: Figure S2 — Total bacterial numbers at day 21. Total cell numbers normalized to the volume of ambient water and oil aggregates per bottle (see text for details). Error bars represent standard deviations of 10 counting fields; note that the standard deviations from average aggregate-associated cell numbers were low, ranging between 0.1% and 0.4%. Letters indicate results from one-way ANOVA followed by the Bonferroni-Holmes test (capital letters: ambient water; small letters: aggregates). Cell numbers with the same letter are statistically indistinguishable. Asterisks indicate significant differences among ambient water and aggregate-associated cell numbers per bottle based on Student's t-test at *p<0.05, **p<0.01, and ***p<0.001. (TIF) [file pone.0034816.s002.tif]

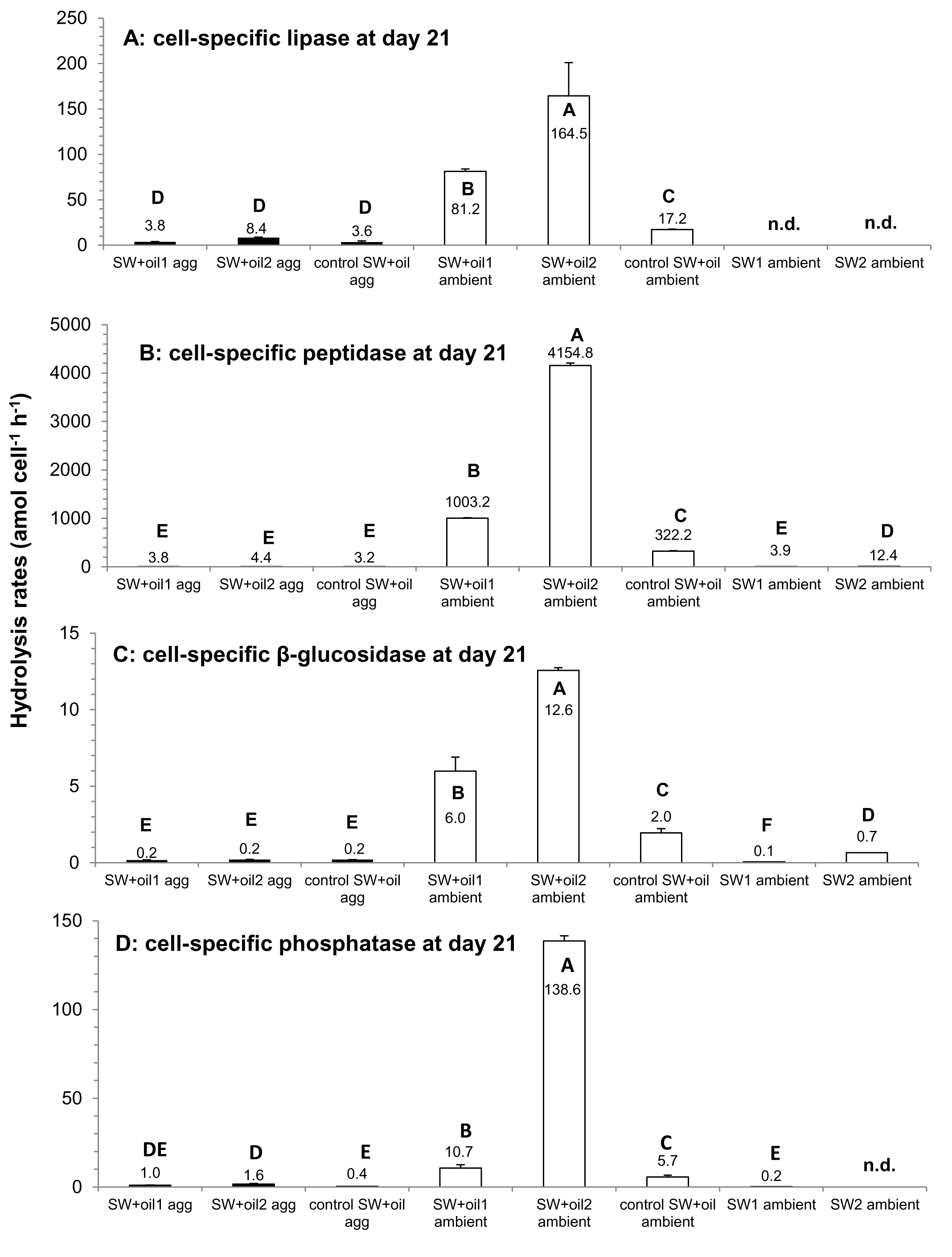

Supplement: Figure S3 — Cell-specific enzyme activities in ambient waters and oil aggregates at day 21. Hydrolysis rates (n = 3 ± standard deviation) on a cell-specific basis. Letters indicate results from one-way ANOVA followed by the Bonferroni-Holmes test. Rates with the same letter are statistically indistinguishable. Data labels are average hydrolysis rates. Note the different scales on the y-axis; n.d. means not detectable. (TIF) [file pone.0034816.s003.tif]

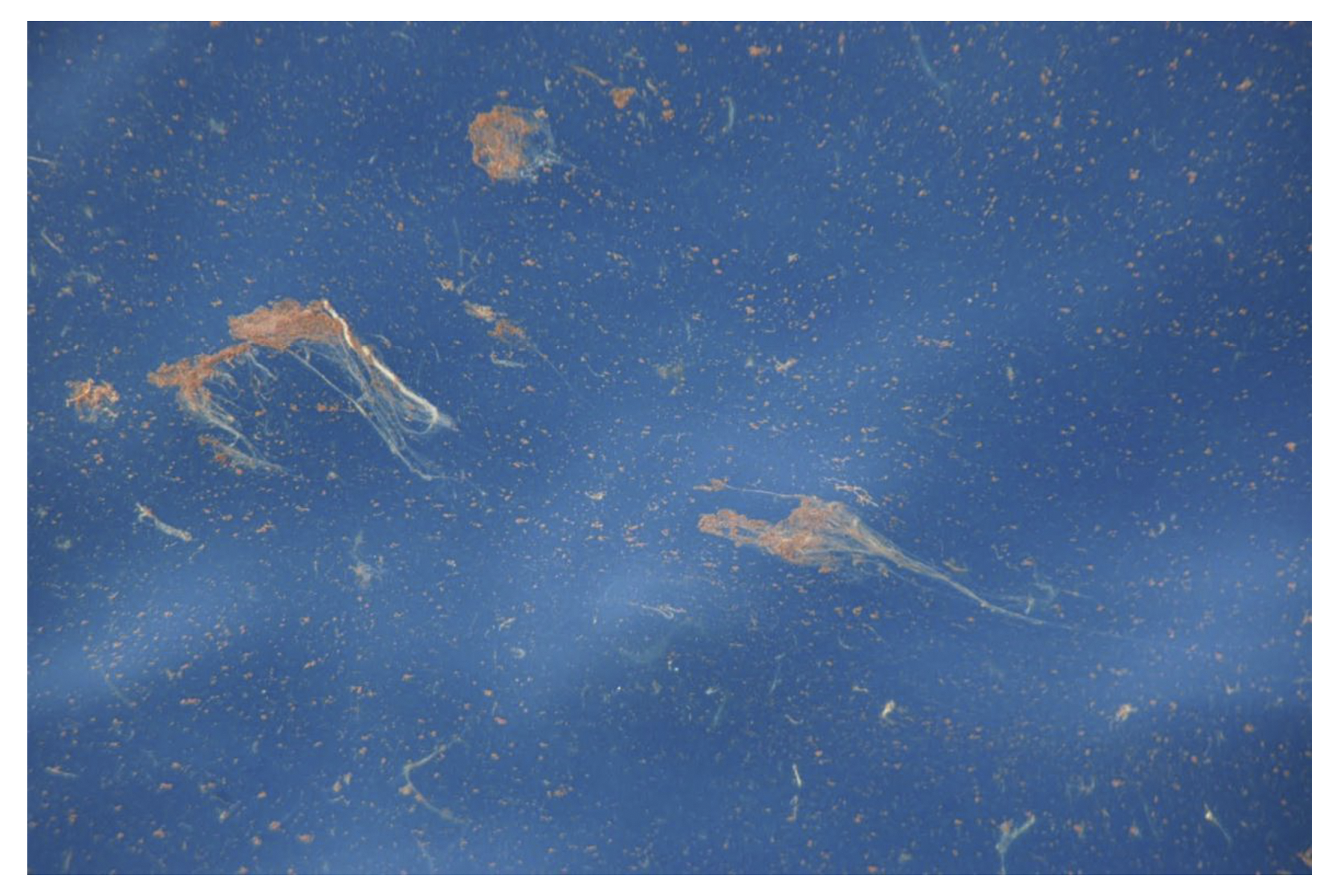

Supplement: Figure S4 — Floating oil aggregates in surface water near the spill site. Photo taken by Arne-R. Diercks (NIUST) on May 11, 2010, onboard the R/V Pelican. (TIF) [file pone.0034816.s004.tif]
