# Supplementary material for: Microbial Activities and Dissolved Organic Matter Dynamics in Oil-Contaminated Surface Seawater from the Deepwater Horizon Oil Spill Site
Source: PLoS One. 2012 Apr 11;7(4):e34816. doi: 10.1371/journal.pone.0034816 (PMC3324544; doi:10.1371/journal.pone.0034816)
Supplement: Methods S1 — Detailed description of enzyme activity, cell counts and CDOM measurements. (PDF) [file pone.0034816.s005.pdf]

Methods S1

Enzyme activity measurements. Hydrolysis rates of MUF- and MCA-substrates were determined by dispensing 0.5 ml of bottle water into disposable methacrylate cuvettes preloaded with a single substrate analog. Care was taken to avoid transferring oil from the 50-ml tubes that had oil contaminated seawater (SW+oil and control SW+oil), as oil residues tended to stick to the outside of the pipette tips. Therefore, we first pipetted 0.5 ml of the subsamples into 1.5-ml microcentrifuge tubes; only if no visible oil was present in the latter tubes, we transferred the sample (using a clean pipette tip) into the incubation cuvettes. Samples were incubated in triplicate for two hours at 25 °C in the dark. Initial tests revealed that this incubation time was sufficient to yield significant changes in fluorescence for all of the substrates. Fluorescence was measured in samples containing 1.5 ml borate buffer (50 nM; pH 8.5) using a Turner Biosystems TBS-380 fluorometer, with excitation/emission channels set to “UV” (365 nm excitation, 440-470 nm emission).

Oil aggregates were incubated in triplicates under the same conditions as described above, except that 0.5 ml of aggregates diluted 0.5 ml filtered and autoclaved seawater were incubated in cuvettes preloaded with a single substrate analog. This was necessary because samples were filtered through a 0.2-µm syringe filter (final volume of the filtrate: 0.5 ml) prior to fluorescence measurements to avoid any interference caused by the presence of particulate matter. Fluorescence measurements were calibrated using MUF and MCA standards dissolved in uncontaminated and oil-contaminated water. Note that all hydrolysis rates reported here are potential rates, since they were measured with

an externally-added substrate that competes with naturally-occurring substrates of unknown concentration for enzyme active sites.

Microbial cell abundance. One milliliter of ambient water per time point was stained with 4’, 6-diamidino-2-phenylindole (DAPI; 10% v/v) for 10 minutes and filtered through a 0.2 μm GTBP isopore filter (Millipore, Billerica, MA). Thereafter, samples were fixed in 4% paraformaldehyde (PFA in phosphate buffered saline) for 25 minutes. After washing with sterile water and PBS, filters were placed on glass microscope slides with antifade (Citifluor Ltd, UK) and stored at -20 °C until the end of the incubation. For cell enumeration in oil aggregates, one milliliter of aggregates diluted in filtered and autoclaved seawater was further diluted (1:100) and fixed with 2% PFA for 24 hours, and incubated with 0.001M sodium pyrophosphate for 15 minutes ($Na_4P_2O_7$; 1:200 dilution). Samples were sonicated (6 x 5-s bursts at 30 Watts) with a Sonic Dismembrator 60 (Fisher Scientific), stained with DAPI, and stored in the dark at -20 °C. For microscopic cell enumeration, duplicate filters per sample were examined under an epifluorescence microscope (Olympus, magnification x1000) equipped with a digital camera (Olympus TH4-100), and cells from ten randomly chosen frames were counted.

CDOM. A 1 cm quartz cuvette was used for all samples and measured absorbance (A) was converted to absorption coefficients (*a*) using Equation 1.

$$a_{(\lambda)} = 2.303 A_{(\lambda)} / L \qquad (1)$$

L is the pathlength in meters (0.01 m). CDOM excitation-emission matrix (EEM) fluorescence was measured immediately following absorption measurements on a Varian Eclipse spectrofluorometer. Excitation wavelengths ranged from 220 to 500 nm at 5 nm increments, while emission wavelengths were sampled every 2 nm from 240 to 600 nm.

EEM fluorescence intensities were corrected for Rayleigh and Raman scattering, Milli-Q water blank-subtracted, and instrument bias in excitation and emission prior to correction for any inner-filtering effect [1]. Appropriate corrections for samples that were diluted were performed prior to a final calibration of fluorescence in each EEM to quinine sulfate equivalents (ppb QSE; [2]).

References

1. Stedmon CA, Bro R. (2008) Characterizing dissolved organic matter fluorescence withparallel factor analysis: A tutorial. Limnol Oceanogr: Methods 6: 572-579.

2. Lawaetz AJ, Stedmon CA. (2009) Fluorescence intensity calibration using the raman scatter peak of water. Appl Spectrosc 63(8): 936-940.
